# Potential use of low-copy nuclear genes in DNA barcoding: a comparison with plastid genes in two Hawaiian plant radiations

**DOI:** 10.1186/1471-2148-13-35

**Published:** 2013-02-09

**Authors:** Yohan Pillon, Jennifer Johansen, Tomoko Sakishima, Srikar Chamala, W Brad Barbazuk, Eric H Roalson, Donald K Price, Elizabeth A Stacy

**Affiliations:** 1 Tropical Conservation Biology and Environmental Science, University of Hawai‘i at Hilo, 200 West Kawili Street, Hilo, HI, 96720, USA; 2 Department of Biology, University of Florida, Gainesville, FL, 32611, USA; 3 Genetics Institute, University of Florida, Gainesville, FL, 32610, USA; 4 School of Biological Sciences, Washington State University, 339 Abelson Hall, Pullman, WA, 99164-4236, USA

**Keywords:** Adaptive radiation, Island biogeography, Lobeliads, Next-generation sequencing, Progression rule, Single-copy nuclear genes

## Abstract

**Background:**

DNA barcoding of land plants has relied traditionally on a small number of markers from the plastid genome. In contrast, low-copy nuclear genes have received little attention as DNA barcodes because of the absence of universal primers for PCR amplification.

**Results:**

From pooled-species 454 transcriptome data we identified two variable intron-less nuclear loci for each of two species-rich genera of the Hawaiian flora: *Clermontia* (Campanulaceae) and *Cyrtandra* (Gesneriaceae) and compared their utility as DNA barcodes with that of plastid genes. We found that nuclear genes showed an overall greater variability, but also displayed a high level of heterozygosity, intraspecific variation, and retention of ancient alleles. Thus, nuclear genes displayed fewer species-diagnostic haplotypes compared to plastid genes and no interspecies gaps.

**Conclusions:**

The apparently greater coalescence times of nuclear genes are likely to limit their utility as barcodes, as only a small proportion of their alleles were fixed and unique to individual species. In both groups, species-diagnostic markers from either genome were scarce on the youngest island; a minimum age of ca. two million years may be needed for a species flock to be barcoded. For young plant groups, nuclear genes may not be a superior alternative to slowly evolving plastid genes.

## Background

DNA barcoding is a recent technique that employs one or a few short, universal DNA regions to place sampled individuals into named species and to identify individuals as belonging to putatively undescribed species (http://www.barcodeoflife.org/). DNA-based identification promises a range of applications, including identification of organisms at cryptic life stages (e.g., seeds, seedlings, larvae), source identification of plant or animal parts (e.g., plant foodstuffs, herbal medicines, meats and furs from CITES-protected species), forensics, and surveys of poorly known biological communities e.g., tropical rainforests, deep-sea communities, microbial communities [1].

Although DNA barcoding of animals using mitochondrial genes has been done with high success [2], plants have proven to be somewhat recalcitrant to DNA barcoding. The low sequence variation in the plant mitochondrial genome has led to a search for alternative universal DNA barcodes for plants, which has proven difficult [3]. Most genes tested as universal plant DNA barcodes are within the plastid genome, and a small number of them are becoming increasingly popular [4]. A single gene is unlikely to provide enough resolution to differentiate all plant species [5], yet six plastid genes in combination still fail to discriminate all species within the genus *Crocus*[6]. Other candidates include nuclear ribosomal genes [7]; however, their utility as DNA barcodes may be limited by incomplete concerted evolution, fungal contamination, and amplification failure [3]. Although DNA barcoding using multiple genes has proven successful with high resolution for phylogenetically diverse communities, e.g., Panamian trees (98% species discrimination, [8]), Mesoamerican orchids and Kruger National Park trees (> 90% species identification, [9]), but see Gonzalez et al. (< 70% species identification, [10] for Amazonian trees), barcoding studies of single clades have had limited success, e.g., 43.5% species discriminated in Bromeliaceae [11], and 32% in *Fraxinus*[12]. Alternative candidates for DNA barcodes are low-copy nuclear genes, which have received little attention (i.e., [13]). Problems expected with such genes include the design of universal primers, gene duplications, recombination, allopolyploidy and heterozygosity [3].

The native flora of Hawai‘i boasts extreme endemism (89% for angiosperms [14]) and offers a unique opportunity to evaluate DNA barcoding on species and communities of different ages. The Hawaiian flora is a rich but young assemblage, with the majority of lineages originating on the main islands within the past five million years [15]. Hawaii’s main islands are part of a broader volcanic chain and span a natural age gradient from 0.5 to 5 my [15]. Although many of Hawaii’s endemic plant lineages span the main islands, most species are restricted to a single island [16], and their maximum ages can be set to the age of the island on which they occur. As such, the Hawaiian flora allows estimation of a species-age or a community-age threshold below which DNA barcoding fails to delineate species.

To our knowledge, DNA barcoding has not been attempted on the Hawaiian flora, and, plastid genes (the most popular DNA barcodes) have comparatively been little used for phylogenetic studies of Hawaiian radiations, presumably due to a near absence of variation in these genes. Instead, most studies have used ribosomal genes [17-21] or in a few cases low-copy nuclear genes [22,23], finding moderate levels of variation at these genes. One of the notable exceptions is the phylogeographic study of *Metrosideros* by Percy et al. [24] based on 10 plastid genes; nonetheless, these genes failed to fully resolve the evolutionary history of the genus within the Hawaiian Islands. The availability of DNA barcodes for the Hawaiian flora is particularly desirable as many native species are difficult to distinguish through vegetative characters alone, many are threatened by extinction, and hybridization appears to be common.

Our study focused on two plant genera of the Hawaiian Islands: *Clermontia* (Campanulaceae) and *Cyrtandra* (Gesneriaceae). *Clermontia* is an endemic genus of rainforest shrubs that are either epiphytic or terrestrial with bird-pollinated flowers that show great variation in flower morphology among species. The genus belongs to the Hawaiian lobeliads, the largest Hawaiian radiation [25], and comprises 22 species [14], most of which are found on the youngest islands of Maui and Hawai‘i (Big Island). *Cyrtandra* is a genus of understorey shrubs with somewhat uniform, white and probably insect-pollinated flowers and great variation in vegetative characters, although the adaptive significance of the latter is generally not clear. The genus comprises 53 species [14], all endemic, more or less evenly distributed among the main islands, with O‘ahu having the greatest diversity. In both genera, most species are restricted to a single island, and numerous cases of hybridization have been reported. Furthermore, circumscription of species with multiple-island distributions is often disputed. The estimated crown ages for *Clermontia* and Hawaiian *Cyrtandra* are 3.2 My [25] and 5.2 My [26], respectively.

In this study, we evaluate the potential of low-copy nuclear genes as DNA barcodes in *Clermontia* and *Cyrtandra*, and discuss some of their advantages and disadvantages compared to frequently used plastid genes. We did not attempt to identify universal barcodes, but rather conducted a pilot study to see how such markers would be informative.

## Results

### Variation within single-copy nuclear genes vs. plastid genes

**Figure 1:**
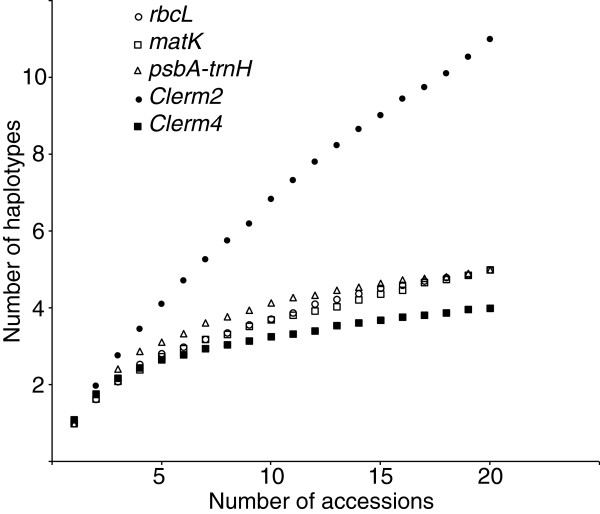
**Accumulation curves for haplotypes in plastid and nuclear genes in *Clermontia*.** Accumulation curves for haplotypes in three plastid (*rbcL*, *matK*, *psbA*-*trnH*) and two nuclear (*Clerm2*, *Clerm4*) genes in *Clermontia*.

**Figure 2:**
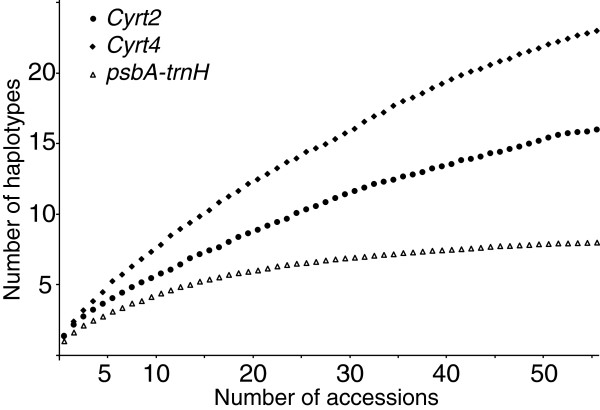
**Accumulation curves for haplotypes in plastid and nuclear genes in *Cyrtandra*.** Accumulation curves for haplotypes in one plastid (*psbA*-*trnH*) and two nuclear (*Cyrt2*, *Cyrt4*) genes in *Cyrtandra*.

Although relatively short in length, the nuclear genes generally exhibited a greater number of variable sites than plastid genes and had a percentage of variable sites up to several fold that of plastid genes (Tables 1 &2). Heterozygosity was common in nuclear genes (Additional files 1 &2): 25% of the individuals at *Clerm2,* 5.5% at *Clerm4*, 34.4% at *Cyrt2* and 36.1% at *Cyrt4*. Accumulation curves (Figures 1 and 2) for plastid genes showed that in both genera, a plateau was reached and that most of the haplotype diversity present in each group was captured in our study. In contrast, with the exception of *Clerm4* which was especially short (172 bp), the slopes of the nuclear haplotype accumulation curves showed no inflexion, indicating that many more haplotypes could be found with further sampling. Limited variation and especially low numbers of differences between haplotypes did not allow detection of recombination in any nuclear genes.

**Table 1:** **Variability and properties of the markers (three plastid, two nuclear) used in *Clermontia***

	***rbcL***	***psbA-trnH***	***matK***	**Combined plastid**	***Clerm2***	***Clerm4***
Length, excluding primers	533	439	837-858	1829-1850	556	172
Number of variable sites (number of indels)	5	5	4 (1)	14 (1)	10	3
Percent of variable sites, excluding indels	0.90	1.14	0.005	0.008	1.80	1.74
Percent of “ghost” haplotypes	16.7	16.7	16.7	56.3	0	0
Ramification index	0	0.2	0	0.13	0.58	0.25

“Ghost” alleles are intermediate alleles that were not recovered in any of the accessions sampled. The ramification index (I) was calculated as follows: I = 1 – (longest distance between two haplotypes/total length of the network).

**Table 2:** **Variability and properties of the markers used in *Cyrtandra***

	***psbA-trnH***	***Cyrt2***	***Cyrt4***
Length, excluding primers	388-403	314	291
Number of variable sites (number of indels)	5 (2)	19	21
Percent of variable sites, excluding indels	1.29	6.05	7.21
Percent of “ghost” haplotypes	11.1	0	10
Ramification index	0.5	0.66	0.61

“Ghost” alleles are intermediate alleles that were not recovered in any of the accessions sampled. The ramification index (I) was calculated as follows: I = 1 – (longest distance between two haplotypes/total length of the network).

**Figure 3:**
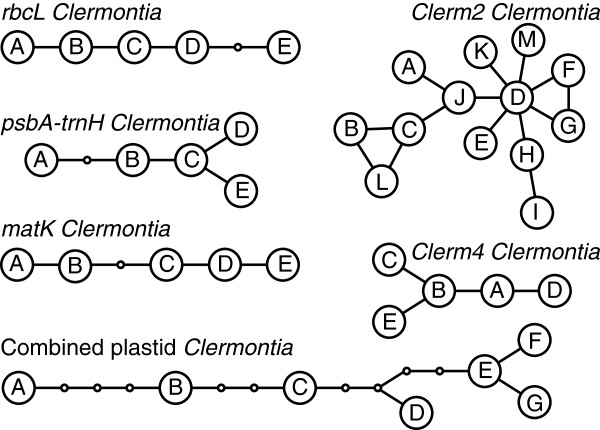
**Haplotype networks for plastid and nuclear genes in *Clermontia.*** Haplotype networks for plastid (*rbcL*, *matK*, *psbA*-*trnH*) and nuclear (*Clerm2*, *Clerm4*) genes in *Clermontia*. The small empty circles indicate “ghost” alleles (intermediate alleles that were not recovered in any of the accessions sampled).

**Figure 4:**
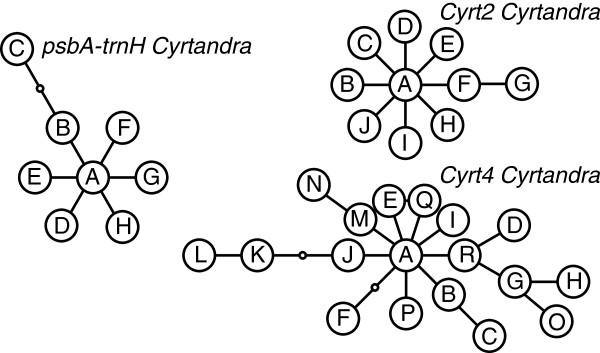
**Haplotype networks for plastid and nuclear genes in *Cyrtandra.*** Haplotype networks for plastid (*psbA*-*trnH*) and nuclear (*Cyrt2*, *Cyrt4*) genes in *Cyrtandra*. The small empty circles indicate “ghost” alleles (intermediate alleles that were not recovered in any of the accessions sampled).

In *Clermontia*, where haplotype networks for plastid and nuclear genes could be contrasted (i.e., haplotypes were available for two genes of each type), networks differed in their structure (Figure 3). With a minor exception, the plastid gene networks for *Clermontia* were strictly linear (I = 0, Table 1), whereas the network for *Clerm2* was star-like with the occurrence of loops. The network for the short nuclear gene, *Clerm4*, had only five haplotypes and was neither strictly linear nor starlike (Figure 3). Ramification indices for the two nuclear gene networks were greater than those for the three plastid genes. Each plastid gene network contained a single intermediate haplotype that was not recovered in any accession (“ghost” haplotype); there was no such haplotype in the nuclear datasets. For *Cyrtandra*, the single plastid gene and two nuclear genes showed highly ramified haplotype networks (Figure 4), but the ramification indexes indicated a greater degree of reticulation in the networks of the two nuclear genes compared to the plastid gene. There was one ghost haplotype (out of 9) in the plastid network, none in the nuclear *Cyrt2* network, and two (out of 21) in the *Cyrt4* network.

### Taxonomic distribution of genetic diversity

For *Clermontia*, the plastid and nuclear genes revealed species-diagnostic haplotypes (here defined as haplotypes unique to a single species and found in all individuals of that species) for 18% and 12% of the sampled taxa, respectively. Each of the three plastid genes had two species-diagnostic haplotypes, and the combined dataset produced diagnostic haplotypes for three species: *Cl. fauriei* (Kaua‘i), *Cl. oblongifolia* (O‘ahu) and *Cl. kakeana* (O‘ahu and Maui; figure five). In nuclear genes, *Clerm2* yielded a single species-diagnostic haplotype for *Cl. fauriei*, and *Clerm4* a single species-diagnostic haplotype for *Cl. oblongifolia*. Several species possessed multiple haplotypes for a given nuclear gene, with up to six in *C. arborescens* for *Clerm2*. Species-diagnostic haplotypes that differ by more than one substitution from others (i.e., interspecific gap) were found only for *Cl. fauriei* and *Cl. oblongifolia* in the combined plastid dataset. Conflicts were present in the genetic identity of *Cl. singuliflora*; this species grouped with *Cl. peleana* in the plastid dataset but was closer to *Cl. parviflora* and allies in the nuclear datasets.

For *Cyrtandra*, the plastid and nuclear genes revealed species-diagnostic haplotypes for 20% and 10% of the sampled taxa, respectively. The *psbA*-*trnH* dataset yielded diagnostic haplotypes for four species: *Cy. longifolia* (Kaua‘i), *Cy. wawrae* (Kaua‘i), *Cy. lydgatei* (Maui) and *Cy*. *paludosa* (Kaua‘i, O‘ahu and Hawai‘i; figure six). In contrast, the nuclear *Cyrt2* gene revealed just one species-diagnostic haplotype (*Cy. longifolia*), and the *Cyrt4* gene revealed diagnostic haplotypes for *Cy. grayi* (Maui), that were not distinguished by either of the above markers. Only *Cy. platyphylla* had multiple plastid haplotypes (one on Maui and one on Hawai‘i Island), whereas 12 species had multiple haplotypes in the *Cyrt2* dataset and 9 in the *Cyrt4* dataset. A species-diagnostic haplotype that differs by more than one substitution was found for just one species, *Cy. longifolia*, in the *psbA-trnH* dataset.

### Geographic distribution of haplotype diversity

Across genes and genera, there appeared to be a positive relationship between haplotype diversity and island age (Additional file 3). In *Clermontia*, plastid haplotypes occurred on two islands at most, whereas some nuclear haplotypes were found on three islands. In *Cyrtandra*, for which haplotype variation was greater, there were several haplotypes at each of the three genes that occurred on three or four islands.

**Figure 5:**
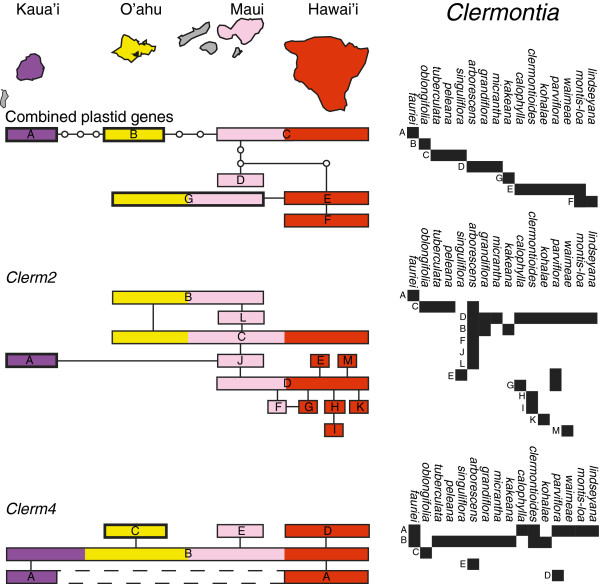
**Geographic and taxonomic distributions of haplotypes in *Clermontia*.** The combined plastid dataset includes data from *rbcL*, *matK* and *psbA*-*trnH*; *Clerm2* and *Clerm4* are two nuclear loci. The left-hand side shows the haplotype network and the geographical distribution of alleles. Empty circles indicate “ghost” alleles (intermediate alleles that were not recovered in any of the accessions sampled). Thickened rectangles indicate species-diagnostic haplotypes. The right-hand side shows the taxonomic distribution of the haplotypes.

**Figure 6:**
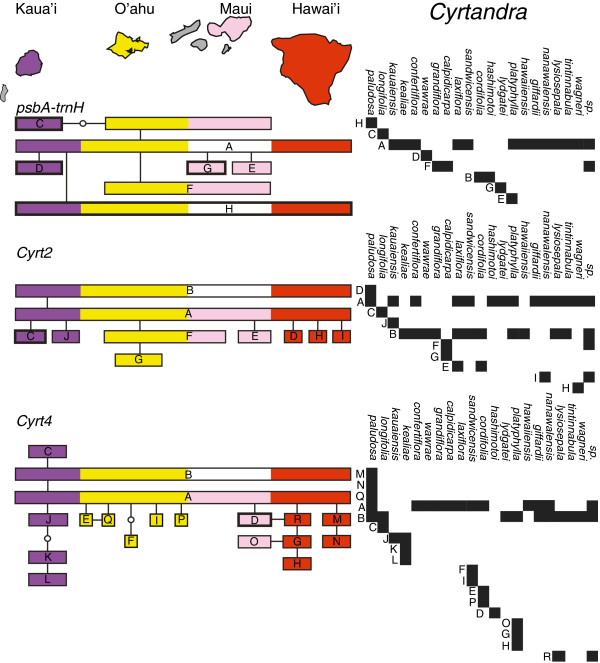
**Geographic and taxonomic distributions of haplotypes in *Cyrtandra.****psbA*-*trnH* is a plastid locus and *Cyrt2* and *Cyrt4* are two nuclear loci. The left-hand side shows the haplotype network and the geographical distribution of alleles. Empty circles indicate “ghost” alleles (intermediate alleles that were not recovered in any of the accessions sampled). Thickened rectangles indicate species-diagnostic haplotypes. The right-hand side shows the taxonomic distribution of the haplotypes.

In contrast to the nuclear genes that showed almost no geographic structure, plastid gene networks aligned to a high (*Clermontia*) or low (*Cyrtandra*) degree with the geographic order of islands (Figures 5 &6). In plastid gene networks for *Clermontia*, haplotypes from Kaua‘i were on one end, and haplotypes from Hawai‘i Island were generally on the other. In the *psbA-trnH* datast of *Cyrtandra*, the most isolated haplotype (C) was from Kaua‘i. In nuclear datasets in contrast, no geographic structure could be detected in the star-like networks in either genus; the nuclear genes generally possessed one or two common haplotypes that were found on multiple islands. Nevertheless, the branch bearing the haplotypes J, K and L of the *Cyrt4* network was restricted to Kaua‘i. For both genera, the greatest resolution of species was possible for the oldest island of Kaua’i (e.g., *Cl. fauriei* was unique at multiple genes). Removing Kaua’i species from the analysis eliminated most of the observed geographical structure and almost all of the species-diagnostic markers.

## Discussion

### Greater coalescence times in nuclear genes

Despite their modest lengths, the nuclear genes examined in this study were highly variable relative to plastid genes, most likely due to their greater coalescence times. The short lengths of the nuclear regions (between 172 and 556 bp) resulted from the difficulty of finding genes with long exons that would allow direct sequencing in all accessions. Nevertheless, the high percentage of variable sites in these genes compared to plastid genes allowed detection of a greater number of haplotypes. Furthermore, the lack of plateau in the accumulation curves for three of the four nuclear genes indicated that a significant number of haplotypes were not captured in our samples.

The greater variability of nuclear loci and many other differences with plastid genes can be explained by the greater coalescence times of nuclear genes compared to organelle genes [27]. Because of the larger effective population sizes of nuclear genes (two alleles per individual) compared to plastid genes (a single allele per individual), genetic drift is less influential, ancestral haplotypes are maintained for longer periods of time, and fixation of novel mutations in nuclear genes requires longer time periods, perhaps three times as long as required for plastid genes [27]. In the nuclear genes examined in this study, ancestral haplotypes are still present at the centre of the star-like networks and are also often abundant and widespread across species, thus contributing to high intraspecific polymorphism. Particularly in *Cyrtandra*, high polymorphism was maintained in nuclear genes in many species, and the multiple alleles present are probably much older than their corresponding species or even the islands on which the species are found. Similarly, genetic diversity within islands was greater for nuclear genes relative to plastid genes, and the elapse time between the formation of two consecutive Hawaiian islands is probably too short to allow for the fixation of a single allele within each island. Thus, ancestral alleles are spread through these populations during colonization of new islands, resulting in the presence of several common nuclear haplotypes across the archipelago from Kaua‘i to Hawai‘i. In plastid genes, in contrast, intraspecific polymorphism was rare. Rather, the plastid gene networks revealed that several intermediate haplotypes have been lost (particularly in *Clermontia*), most likely through drift. In summary, the comparison between nuclear and plastid genes reveals that the plastid genes possess a greater number of species-diagnostic haplotypes, some of which were distinct by more than one substitution, allowing delineation of a larger number of species with greater confidence.

### Is there a species-age threshold below which DNA barcoding fails?

With its ~5-million-year chronosequence of islands of known ages, large number of island-endemic species, and generally positive relationship between island age and species age within many taxonomic groups (i.e., the progression rule, [28]), Hawai’i offers a unique opportunity to examine the evolutionary timescale over which DNA barcoding works. The progression rule [28,29] assumes that most native plant and animal lineages colonized Hawai’i by way of the oldest main island of Kaua’i (4.7 My, [15]), and then spread to each new island to the east as it formed: O‘ahu (3.0 My), Maui nui (Maui, Molokai‘i, Lana‘i, 2.2 My) and finally Hawai‘i (0.5 My).

Evidence for the progression rule in *Clermontia* is clear. A previous phylogenetic analysis based on plastid genes [25] suggested that the single Kaua‘i species *Cl. fauriei* is sister to all other Hawaiian *Clermontia*, and this same pattern was recovered with an extended sampling of genes and species (Y. Pillon et al. unpublished). *Clermontia’s* putative sister group, *Cyanea*, also probably originated on Kaua‘i [30]. Further, our combined plastid network revealed a linear distribution of haplotypes congruent with the progression rule, from one end to the other: Kaua‘i, O‘ahu and Maui-Hawai‘i. Two independent colonizations of Hawai‘i from Maui are indicated, and the single haplotype of *Clermontia kakeana* (O‘ahu, Maui nui), is nested within a clade from Hawai‘i, thus indicating westward migration between islands. This combined evidence strongly indicates that *Clermontia* originated on Kaua‘i and then colonized the younger islands roughly in the order of their formation. The nuclear *Clerm2* gene network, despite its abundant reticulation and low divergence between haplotypes, is weakly consistent with the above pattern.

Although the progression rule is not clear in *Cyrtandra*[26], there are some lines of evidence that support an origin for this group on Kaua‘i. All three genes revealed common haplotypes that were found on all islands. However, the most divergent haplotypes for both *Cyrt4* and *psbA-trnH* were found on Kaua’i. Furthermore, haplotype diversity increased with island ages (Additional file 3). Within all three genes, occurrence of several haplotypes across most or all islands suggests that each island has been colonized multiple times by *Cyrtandra*.

Both plant groups therefore appear to show at least a rough association between island age and species age, and this study revealed more species-diagnostic haplotypes on the oldest island of Kaua’i than on any other island. The single *Clermontia* species from Kaua’i, *Cl. fauriei*, was genetically distinct at five of six loci. Each of three *Cyrtandra* species from Kaua’i had a unique diagnostic haplotype in one or more markers: *Cy*. *longifolia*, *Cy*. *paludosa* and *Cy. wawrae*. When investigated with additional markers, all species of *Cyrtandra* on Kaua’i can be distinguished (Y. Pillon et al. unpublished). In contrast, no species endemic to the youngest island of Hawai’i had species-diagnostic markers. In most cases, species from this island displayed one of the common haplotypes, associated in some cases with one rare haplotype. Genetic diversity was not much greater on Kaua’i than on Hawai’i Island, but it seems that genetic drift has had more time to sort haplotypes among species on Kaua’i. In *Cyrtandra*, we did not find any species-diagnostic markers on the second-oldest island of O’ahu (albeit the most species rich) or Hawai’i, but found two species-diagnostic markers on Maui. Our sampling of *Cyrtandra* was most limited on this island, as we collected just three of the ten species there; it is highly possible therefore that sampling of additional species would eliminate some or all of the species-diagnostic markers from this young island. In *Clermontia*, in addition to the well differentiated Kaua’i species, *C. fauriei*, the two species sampled from O’ahu (*Cl. oblongifolia* and *Cl. kakeana*), where the genus is poorly diversified, had species-diagnostic markers. On the younger islands, only *Cl. kakeana* from Maui (but also O’ahu) could be identified through our markers, and none of Hawai’i Island’s species could be distinguished. These results suggest that species from Maui and Hawai’i are still too young to be barcoded. There may be a threshold between 3 and 4.7 million years for *Cyrtandra* and between 2.2 and 3.0 million years for *Clermontia* below which species flocks are too young to be barcoded with the markers currently available.

## Conclusions

A major issue preventing the use of low-copy nuclear genes in DNA barcoding has been the absence of universal primers to amplify genes over a large spectrum of plant taxa. Other issues that are less commonly considered include sequencing of accessions with multiple alleles of different lengths. Discerning alleles in these cases with the common Sanger method will require cloning and therefore a significant increase in cost and labor, although this issue may be circumvented in the future with new high throughput sequencing methods (e.g., [31]). Furthermore, multiple plastid regions are easily combined for analysis because each region has a single allele per individual and no recombination. Our examination of two low-copy nuclear genes for each of two Hawaiian plant genera and their comparison with classic barcoding genes from the plastid genome reveals another major issue for the use of nuclear genes in DNA barcoding: their longer coalescence times. The retention of ancient alleles in young species makes discovery of species-specific markers less likely with such genes in spite of their higher level of variability. Studies of more ancient groups are needed to determine whether this problem is restricted to young species radiations. Lastly, the greater variability of nuclear genes makes them desirable markers for phylogenetic studies, but the high frequency of heterozygotes and long coalescence times (which result in the shared retention of ancient alleles in many species) will likely limit their utility in analyses of closely related species.

## Methods

As part of a parallel study involving the development of single-nucleotide polymorphism (SNP) markers, we obtained a pooled, partial transcriptome library from leaf and flower buds [fixed in RNA later (QIAGEN)] of nine taxa: *Clermontia arborescens, Cl. clermontioides, Cl. fauriei, Cl. kakeana, Cl. kohalae, Cl. parviflora Cl. peleana, Cyrtandra longifolia* and a hybrid *Cy. hawaiiensis* × *calpidicarpa*. RNA, cDNA synthesis and 454 sequencing were carried out at the University of Arizona Genetics Core Lab. 454 adapters, ribosomal RNA, low quality, and low-complexity sequences were removed/trimmed using SeqClean (http://compbio.dfci.harvard.edu/tgi/software/), and each taxon was assembled separately by the TGI Clustering tools (TGICL) [32]. We blasted our data against the 400 most highly expressed genes in *Arabidopsis* (C. Fizames, pers. comm.) in CLC DNA Workbench in order to optimize the probability of identifying a set of genes with high coverage in each of all or most species. We selected loci (generally only a small portion of a gene) that comprised a single, long exon (200 bp) with matches in multiple species, and designed primers with FastPCR for their amplification. The presence of introns was tested by comparison with genomic and cDNA sequences in *Arabidopsis* available at http://www.arabidopsis.org. Introns were avoided because preliminary work in both groups showed that introns commonly contained indels, which in the case of heterozygotes with alleles of different lengths prevented reading of direct sequences. After preliminary trials, two nuclear regions were selected for each genus based on ease of amplification and sequencing (absence of paralogs), and level of variation. We selected *Clerm2* (putative homolog of At1g61520, PSI type III chlorophyll a/b-binding protein, Lhca3*1) and *Clerm4* (At3g26520, gamma tonoplast intrinsic protein 2, TIP2) for *Clermontia*; *Cyrt2* (At2g18020 embryo defective 2296, EMB2296/Ribosomal Protein L2) and *Cyrt4* (At4g13940, S-Adenosyl-L-homocysteine hydrolase, SAHH) for *Cyrtandra*. These nuclear regions were amplified using the following mix: 12.3 μL of H_2_O, 4 μL of Gotaq 5 × Buffer (PROMEGA), 2 μL of MgCl_2_ 25 mM, 0.4 μL of dNTP 1.25 μM, 0.2 μL of each primer 10 μΜ, 0.1 μL of GoTaq Flexi DNA polymerase 5u/μL (PROMEGA) and 0.8 μL of DNA template. The following amplification program was used: 2’ at 94°C, 38 cycles of 1’ at 94°C, 1’ at 61°C (for *Cyrt2* and *Cyrt4*, 63°C for *Clerm2* and *Clerm4*), 1’ at 72°C and a final extension of 5’ at 72°C.

To compare the utility of these nuclear genes and plastid genes as DNA barcodes, we sequenced the most universally accepted barcode loci for plants, *matK* and *rbcL* and the most commonly suggested additional locus, *psbA-trnH*. In *Cyrtandra*, amplification of *matK* was difficult (failed or weak amplification), and direct sequences for *rbcL* were often not clear because of the apparent presence of a pseudogene. The latter may result from a gene transfer to the nuclear or mitochondrial genome, a phenomenon that sometimes occur in angiosperms [33]. Therefore, only *psbA-trnH* was sequenced in *Cyrtandra.*

We sequenced 26 populations of 17 species of *Clermontia* from Kaua‘i, O‘ahu, Maui and Hawai‘i (Big Island), typically 4 accessions per population for each nuclear gene; *C. kakeana* was sampled on both Maui and O‘ahu. We included 20 species of *Cyrtandra* from Kaua‘i, O‘ahu, Maui and Hawai‘i, as well as a few undetermined plants (some possibly undescribed species). We sequenced two accessions from one population for each species; *C. paludosa* and *C. platyphylla* were represented by multiple populations on multiple islands. For plastid genes we also sequenced two accessions per population in *Cyrtandra*, but only a single accession per species in *Clermontia* because of the low variation observed. A larger scale genotyping study indicates that variation in plastid markers within *Clermontia* species is uncommon (Y. Pillon et al. unpublished data).

In heterozygous accessions, two haplotypes were determined by comparison with homozygotes following the procedure of Clark [34]; in a few cases the two haplotypes could not be determined. A network of haplotypes was built using the software TCS [35]. For each gene we determined a ramification index (I) to distinguish networks that were linear (no ramification; I = 0) from those that were star-like (highly ramified; I close to 1). We used the following formula: I = 1 – (longest distance between two haplotypes/total length of the network).

## Competing interest

The authors declare that they have no competing interests.

## Authors’ contributions

YP, DP and ES designed the study. JJ performed fieldwork, YP and TS performed molecular work, EHR, WBB and SR contributed data/analytical tools, YP and ES drafted the manuscript. All authors read and approved the final manuscript.

## Supplementary Material

Additional file 1 ***Clermontia* genotypes and DNA accessions.** List of *Clermontia* accessions with geographical origin, voucher information, genotypes and GenBank accession numbers. Click here for file

Additional file 2 ***Cyrtandra* genotypes and DNA accessions.** List of *Cyrtandra* accessions with geographical origin, voucher information, genotypes and GenBank accession numbers. Click here for file

Additional file 3 **Genotypic diversty in *Clermontia* and *Cyrtandra* across Hawaiian islands after rarefaction.** Click here for file
