# Supplementary material for: Potential use of low-copy nuclear genes in DNA barcoding: a comparison with plastid genes in two Hawaiian plant radiations
Source: BMC Evol Biol. 2013 Feb 9;13:35. doi: 10.1186/1471-2148-13-35 (PMC3605094; doi:10.1186/1471-2148-13-35)
Supplement: Additional file 3 — Genotypic diversty in Clermontia and Cyrtandra across Hawaiian islands after rarefaction. [file 1471-2148-13-35-S3.pdf]

Haplotype diversity as a function of island ages, from oldest to youngest (Kaua‘i, O‘ahu, Maui, Hawai‘i).

Accumulation curves were drawn for each island to remove sample size effects as islands were unevenly sampled. For *Clermontia*, only results for Maui and Hawai‘i are shown as the genus is poorly diversified on the islands of Kaua‘i and O’ahu which had therefore a small sample size.

Clermontia

Cless2

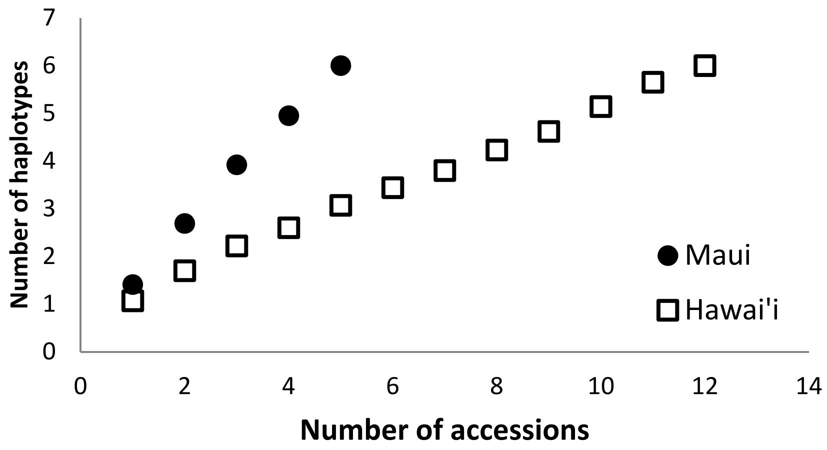

Cless4

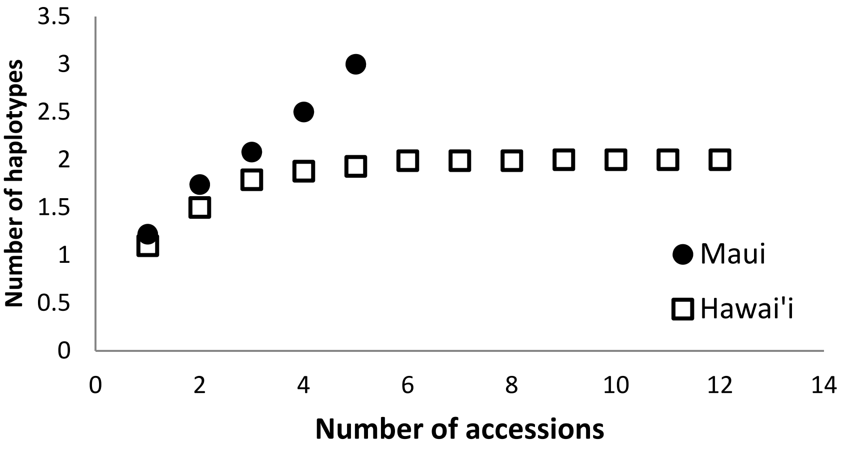

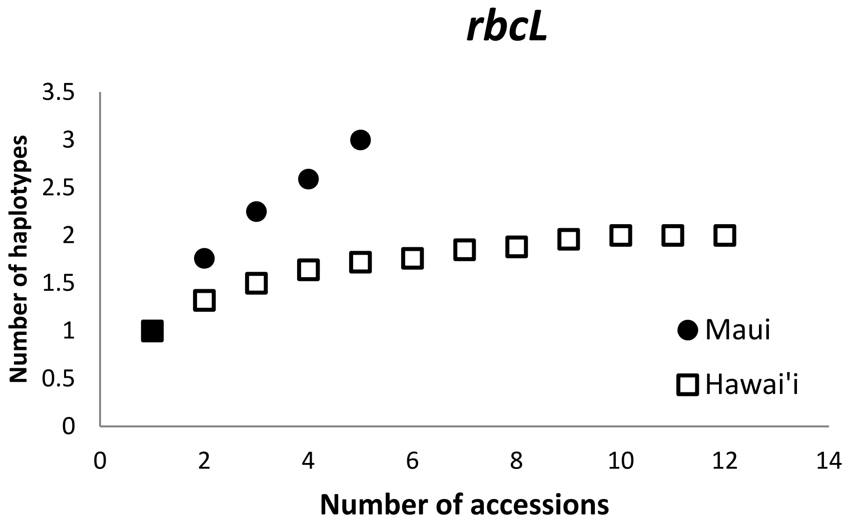
rbcL
Number of haplotypes
Number of accessions
Maui
Hawai'i
0
0.5
1
1.5
2
2.5
3
3.5
0
2
4
6
8
10
12
14

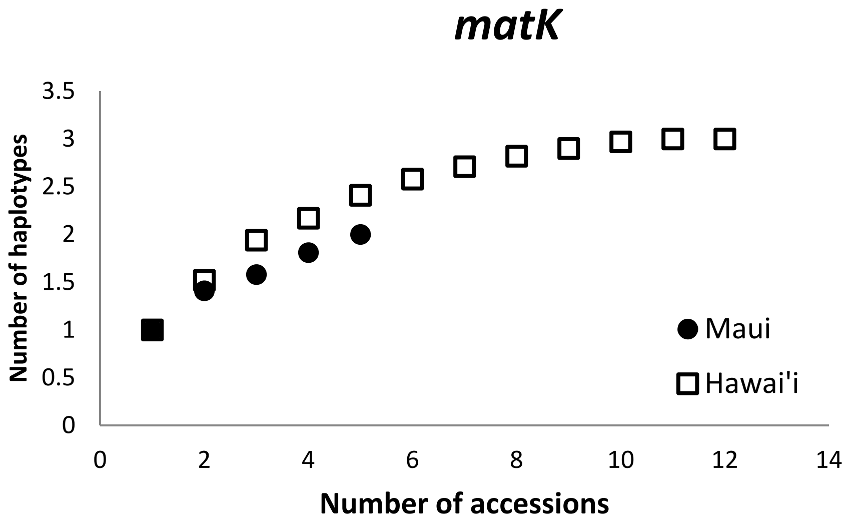
matK
Number of haplotypes
Number of accessions
Maui
Hawai'i
0
0.5
1
1.5
2
2.5
3
3.5
0
2
4
6
8
10
12
14

psbA-trnH

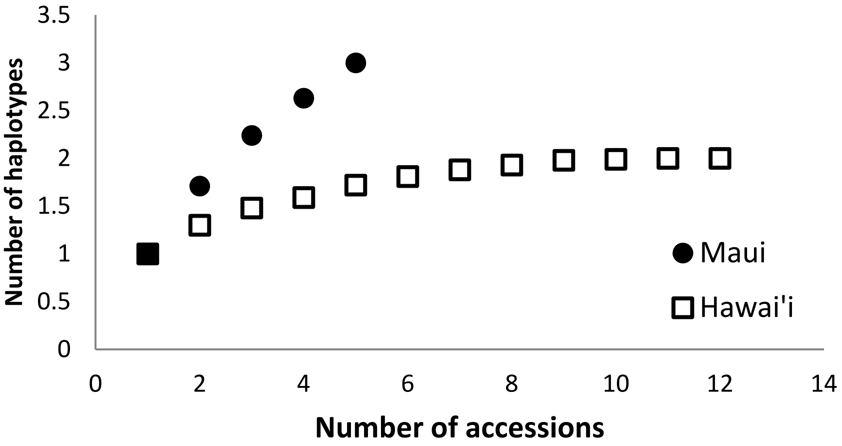

Cyrtandra

Cyss2

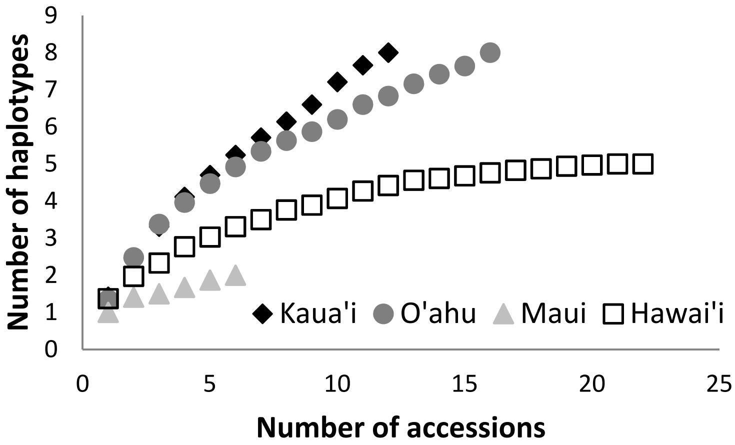

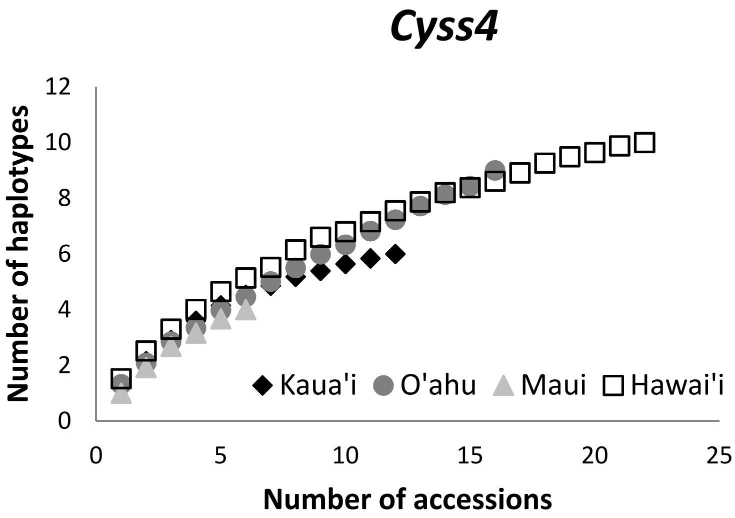
Cyss4
Number of haplotypes
Number of accessions
Kaua'i
O'ahu
Maui
Hawai'i
0
2
4
6
8
10
12
0
5
10
15
20
25

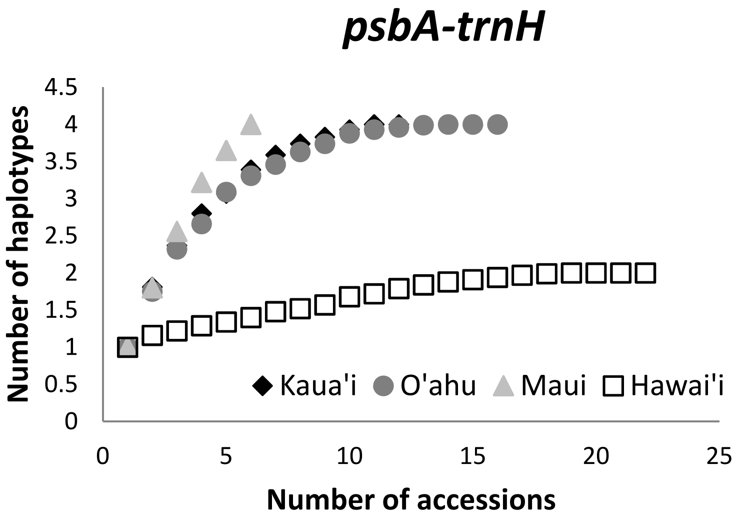
psbA-trnH
Number of haplotypes
Number of accessions
Kaua'i
O'ahu
Maui
Hawai'i
0
0.5
1
1.5
2
2.5
3
3.5
4
4.5
0
5
10
15
20
25
